# Taxonomic revision of the New World genus *Callotillus* Wolcott (Cleridae, Tillinae), with the description of the new genus *Neocallotillus*, and an illustrated key of identification to species

**DOI:** 10.3897/zookeys.617.9970

**Published:** 2016-09-15

**Authors:** Alan F. Burke, Gregory Zolnerowich

**Affiliations:** 1 Department of Entomology, 123 Waters Hall, Kansas State University, Manhattan, KS 66506, USA

**Keywords:** West Indies, North America, Central America, geographic range, taxonomy

## Abstract

The New World checkered beetle genus *Callotillus* Wolcott, 1911 is revised and the new genus *Neocallotillus* established. The subspecies *Callotillus elegans vafer* Wolcott is synonymized with the nominal subspecies, *Callotillus elegans elegans* (Erichson), which is transferred to, and designated as the type species of *Neocallotillus* **gen. n.** as *Neocallotillus elegans* (Erichson, 1847), **comb. n.** Two additional species are transferred from *Callotillus* to the new genus: *Neocallotillus intricatus* (Wolcott & Dybas, 1947), **comb. n.** and *Neocallotillus crusoe* (Wolcott, 1923), **comb. n.**, the latter tentatively and based on Wolcott’s original description. *Callotillus* is now composed of two species: *Callotillus eburneocinctus* Wolcott, 1911 and *Callotillus bahamensis* Vaurie, 1952. All abovementioned species except *Neocallotillus crusoe* are diagnosed and redescribed. In the absence of reference material of *Neocallotillus crusoe*, Wolcott’s original description is transcribed. An illustrated key to species is provided. Characters of taxonomic relevance are illustrated and discussed. Updated distribution maps and locality data for all specimens examined are presented.

## Introduction

*Callotillus* Wolcott is a genus of checkered beetles restricted to the Americas. The group has a wide distribution in the New World, extending throughout much of North and Central America and the West Indies (Fig. 8). Wolcott (1911) erected *Callotillus* for *Tillus elegans* Erichson and *Callotillus eburneocinctus* Wolcott, designating the latter as the type species. In later works, *Callotillus vafer* Wolcott, *Callotillus crusoe* Wolcott, and *Callotillus intricatus* Wolcott & Dybas were described from North America, Puerto Rico and Costa Rica, respectively (Wolcott 1921, 1923; Wolcott and Dybas 1947). Barr (1950) recognized *Callotillus vafer* as a subspecies of *Callotillus elegans* on the basis of apparent discontinuity in the geographic distribution of these species and subtle morphological variations observable throughout their geographic range. Finally, an expeditionary work in the West Indies by Vaurie (1952) retrieved a new species, *Callotillus bahamensis*. The genus *Callotillus* thus presently includes five species and two subspecies: *Callotillus bahamensis* Vaurie, *Callotillus crusoe* Wolcott, *Callotillus eburneocinctus* Wolcott, *Callotillus elegans* Wolcott (including the subspecies *Callotillus elegans elegans* (Erichson) and *Callotillus elegans vafer* Wolcott), and *Callotillus intricatus* Wolcott. In this paper we investigate the species of *Callotillus* within the context of a generic revision. All species comprising the two genera are diagnosed and redescribed, except *Neocallotillus crusoe*, where the original description given by Wolcott (1923) is transcribed. An illustrated key and updated distribution maps are given for all the species treated here.

## Material and methods

The taxonomic sampling consisted of approximately 300 specimens collected in North and Central America, and the West Indies. Male genitalia were dissected if more than one male per species was available. Genitalia extraction and dissection procedures are similar to those outlined by Ekis (1977). Most morphological terminology follows the work of Ekis (1977), Rifkind (1993), and Opitz (2010). Specimens were examined with a Leica MZ 7.5 stereomicroscope. Images were taken and measured using a Leica DFC 500 digital camera, and stacked using the software Zerene Stacker V. 1.04.

Information reflected in the distribution maps was obtained from the locality data of the material examined in this study, the original descriptions of all the species, and the works of Wolcott (1947), Corporaal (1950), Papp (1960), Barr (1975), Rifkind (1996), Leavengood (2008), and Burke et al. (2015).

Material used in this work was borrowed from the following collections:

AMNH
American Museum of Natural History, New York, NY, USA 


BMNH
 British Museum of Natural History Collection, London, UK 


CASC
California Academy of Sciences Collection, San Francisco, California, USA 


CIUM
 Colección de Insectos de la Universidad Autónoma del Estado de Morelos, Centro de Investigación en Biodiversidad y Conservación, UAEM, Mexico 


CNHM
Cincinnati Museum of Natural History, Cincinnati, Ohio, USA 


CNIN
 Colección Nacional de Insectos, Instituto de Biología, UNAM, DF, Mexico 


EMEC
Essig Museum of Entomology, University of California, Berkeley, USA 


FMNH
Field Museum of Natural History, Chicago, Illinois, USA 


FSCA
 Florida State Collection of Arthropods, Gainesville, FL 


EWC
 James E. Wappes Collection, San Antonio, TX, USA 


JNRC
 Jacques Rifkind Collection, Valley Village, CA, USA 


KSUC
 Kansas State University Museum of Entomological and Prairie Arthropod Research Collection, Kansas State University, Manhattan, KS, USA 


MSUC
Michigan State University Insect Collection, East Lansing, Michigan, USA 


NMNH
 Smithsonian National Museum of Natural History, Washington, D.C., USA 


RHTC
 Robert H. Turnbow Jr. Collection, Enterprise, AL, USA 


SEMC
 Snow Museum Entomological Collection, The University of Kansas, Lawrence, Kansas, USA 


TAMU
 Texas A&M Insect Collection, Texas A&M University, College Station, TX, USA 


## Taxonomy

### Key to species of *Callotillus* and *Neocallotillus*

**Table:** 

1	Antennomeres 4-9 of males pectinate (Fig. 3A–C), antennomeres 4-9 of females robust, serrate, (Fig. 3D–F); terminal maxillary palpomeres conical; slender to moderately robust species (Figs 1A–F, 4A)	***Neocallotillus*** (2)
–	Antennomeres 4-9 of males strongly serrate (Fig. 3G–H), antennomeres 4-9 of females moderately serrate (Fig. 3I–J); terminal segments of maxillary palps cylindrical; robust species (Figs 1G–H, 4B)	***Callotillus*** (4)
2	Elytral disc finely punctate, punctations irregularly arranged; median region of each elytron adorned with a transverse, light testaceous to almost whitish, slightly protruding fascia, and one protruding macula on the anterior half of the elytral disc, this macula can be absent in some specimens (Fig. 1A–D)	***Neocallotillus elegans***
–	Elytral disc devoid of punctations; fasciae pattern not as above, elytral disc variously adorned, larger individuals	**3**
3	Each elytron with a testaceous, broad and procurved fascia that initiates at the elytral suture and extends from the median region of the elytral disc to the elytral apex, and small, narrow, moderately oblique marking at the median region of the elytral disc; last third of elytral disc with a semicircular macula (Fig. 1F)	***Neocallotillus crusoe***
–	Each elytron adorned with a pair of elaborate, pale fasciae, and one macula arranged as follows: macula located on the anterior fourth, posterior to the humeral angle; one fascia located on anterior half of elytral disc, and strongly procurved, initiating on elytral suture and ending just before epipleural fold; second fascia moderately oblique, located immediately posterior to the other fascia, initiating at the epipleural fold and not reaching the elytral suture (Fig. 1E)	***Neocallotillus intricatus***
4	Pronotal disc dark testaceous to rufous; anterior half of elytral disc same color as pronotum, posterior half piceous, color transition interrupted by a median, transverse, pale fascia that extends from epipleural fold to elytral suture; median region of elytral disc depressed in lateral view (Fig. 1H)	***Callotillus eburneocinctus***
–	Pronotal integument piceous; elytral disc same color as pronotum, except humeral area testaceous to almost ferrugineous; elytral disc lacking any fascia or maculation; median region of elytral disc feebly depressed in lateral view (Fig. 1–G)	***Callotillus bahamensis***

**Figure 1.**
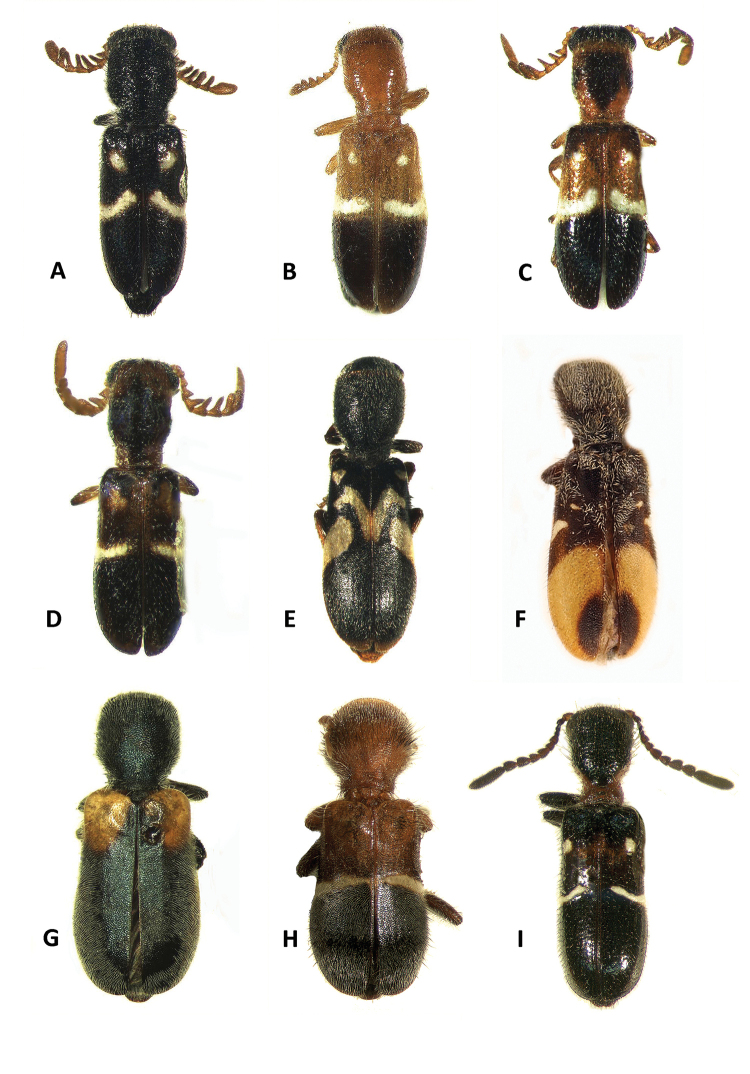
Habitus of: **A** *Neocallotillus elegans* (*Callotillus elegans elegans*) male **B** *Neocallotillus elegans* (*Callotillus elegans vafer*) female **C** *Neocallotillus elegans* (*Callotillus elegans vafer*) male **D** *Neocallotillus elegans* (*Callotillus elegans elegans*) male **E** *Neocallotillus intricatus* (*Callotillus intricatus*) male **F** *Neocallotillus crusoe* (*Callotillus crusoe*) holotype male, (image courtesy of The American Museum of Natural History, New York) **G** *Callotillus bahamensis* male **H** *Callotillus eburneocinctus* male **I** *Barrotillus kropotkini* male.

**Figure 2.**
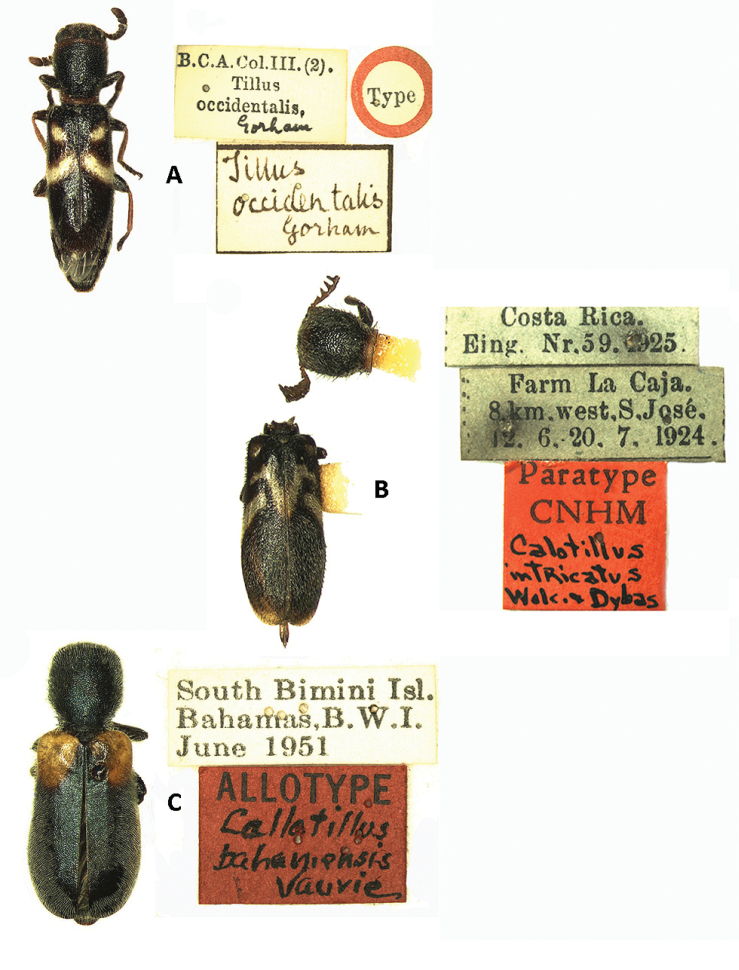
Types and corresponding labels of: **A** *Tillus occidentalis* (Gorham) [*Neocallotillus elegans*], female **B** *Callotillus intricatus* (Wolcott & Dybas) [*Neocallotillus intricatus*], male **C** *Callotillus bahamensis* Vaurie, female.

**Figure 3.**
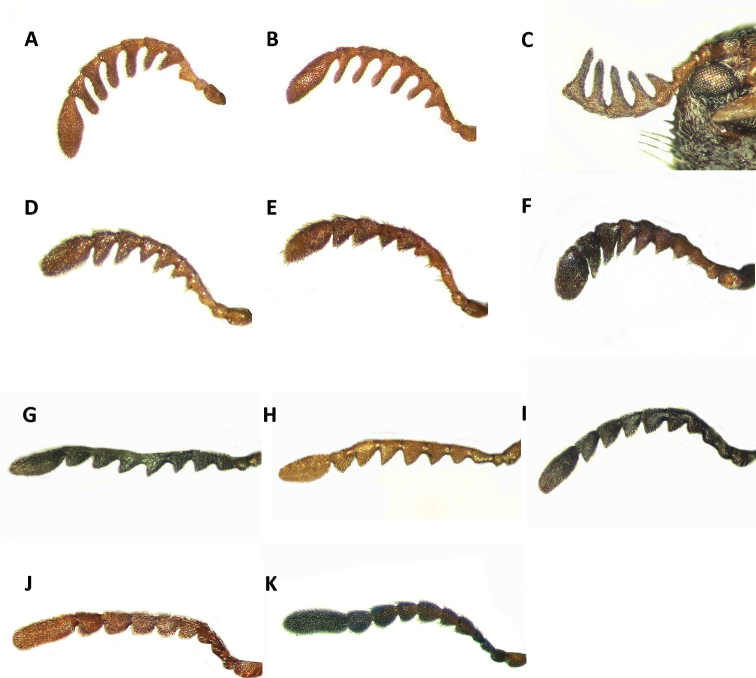
Antennae of: **A** *Neocallotillus elegans* (*Callotillus elegans elegans*) male **B** *Neocallotillus elegans* (*Callotillus elegans vafer*) male **C** *Neocallotillus intricatus* (*Callotillus intricatus*) male holotype **D** *Neocallotillus elegans* (*Callotillus elegans elegans*) female **E** *Neocallotillus elegans* (*Callotillus elegans vafer*) female **F** *Neocallotillus intricatus* (*Callotillus intricatus*) female **G** *Callotillus bahamensis* male **H** *Callotillus eburneocinctus* male **I** *Callotillus bahamensis* female **J** *Callotillus eburneocinctus* female **K** *Barrotillus kropotkini* male.

**Figure 4.**
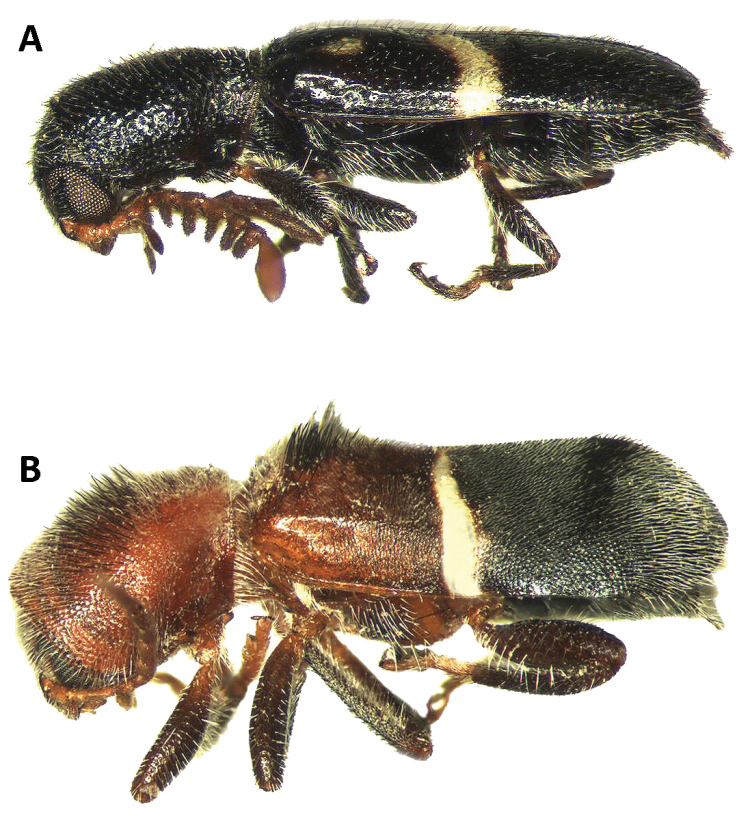
Habitus in lateral view of: **A** *Neocallotillus elegans*, male **B** *Callotillus eburneocinctus*, male.

#### Neocallotillus

Taxon classificationAnimaliaColeopteraCleridae

Burke gen. n.

http://zoobank.org/08D462B4-C0D4-4BE0-82BC-A604244AAAE6

##### Type species.

*Neocallotillus elegans* (Erichson, 1847), here designated.

##### Differential diagnosis.

*Neocallotillus* is most closely related to *Callotillus*. The new genus can be differentiated from *Callotillus* based on the following combination of characters: in *Neocallotillus* species the male has antennomeres 1-2 filiform; the third antennomere is moderately serrate; antennomeres 4-9 are strongly pectinate; and the tenth antennomere is ovoid in shape and laterally depressed (Fig. 3A–C); the length of the tenth antennomere may vary by species. Females have antennomeres 1-3 filiform; the fourth antennomere is moderately serrate; and antennomeres 4-9 are robust, moderately, and gradually increase is size toward the distal end (Fig. 3D–F); the tenth antennomere of females is similar to that of the males. Males of *Callotillus* have antennomeres 1-2 filiform; the third antennomere is moderately serrate; antennomeres 4-9 are strongly serrate and approximately equal in length; and the tenth antennomere is broadly ovoid and about the same length as antennomeres 8-9 combined (Fig. 3G, H). The antennal structure of females is similar to that of males, except antennomeres 4-9 are moderately serrate and the tenth antennomere is cylindrical to moderately ovoid (Fig. 3I, J). Additionally, *Neocallotillus* species are relatively slender and elongate (Figs 1A–E, 4A), while *Callotillus* species are conspicuously more robust (Figs 1F–G, 4B). *Neocallotillus* species lack an elytral swelling present on the anterior third of the elytral disc of *Callotillus* (Fig. 4A–B). *Neocallotillus* also resembles *Barrotillus* Rifkind (Fig. 1I), however, the antenna of *Neocallotillus* is composed of 10 antennomeres (Fig. 3A–F), while the antenna of *Barrotillus* has 11 antennomeres (Fig. 3K). The restricted distribution of *Barrotillus*, recorded only from a confined locality in Honduras, will also serve to separate it from the widely distributed *Neocallotillus*.

##### Description.

Size: 3–7 mm. Color: Light testaceous to dark brown (Fig. 1A–F); costae on elytral disc variously adorned, ranging from light testaceous to brown. Form: small to medium sized individuals; body elongate; elytra subparallel to moderately expanded posteriorly.

Head: Eyes medium sized, moderately taller than wide, conspicuously bulging laterally, strongly emarginate at antennal insertion; diameter of ommatidia small (Fig. 4A); clypeus approximately 3× the width of eye emargination and moderately emarginate medially. Antennae composed of 10 antennomeres; sexual dimorphism observable in antennal shape, where the antennae are moderately pectinate and strongly depressed dorsoventrally in males (Fig. 3A–C), but serrate and somewhat depressed dorsoventrally in females (Fig. 3D–F); tenth antennomere ovoid in both sexes. Labrum elongate, subquadrate; terminal maxillary palp conical, acuminate posteriorly; terminal labial palp securiform.

Thorax: Shape of pronotum scutiform, rounded laterally, narrower than anterior margin of elytra; disc feebly to moderately convex; inconspicuously broader at middle, feebly sinuate, conspicuously constricted on last fourth; anterior depression and antescutellar impression absent. Tibial spur formula 2-2-2, pulvillar formula 4-4-4. Prosternum: Smooth to feebly puncticulate; conspicuously wider than long. Mesoventrite: smooth, puncticulate. Metepisternum visible throughout its length in lateral view, not concealed by elytron. Metaventrite: moderately to strongly convex; variously punctate.

Elytra: Slender to feebly expanded posteriorly, elongate; median region of elytral disc feebly depressed in lateral view; sides subparallel to moderately expanded posteriorly in dorsal view; elytral declivity feebly to moderately gradual; elytral markings always present in various shapes, may be protruding or not.

Legs: Femora smooth, variably vested. Tibiae feebly to moderately rugulose, weakly expanded posteriorly, variously vested. Two tarsal denticles conspicuously separated from each other, inner tarsal denticles trigonal, outer tarsal denticles digitiform.

**Figure 5.**
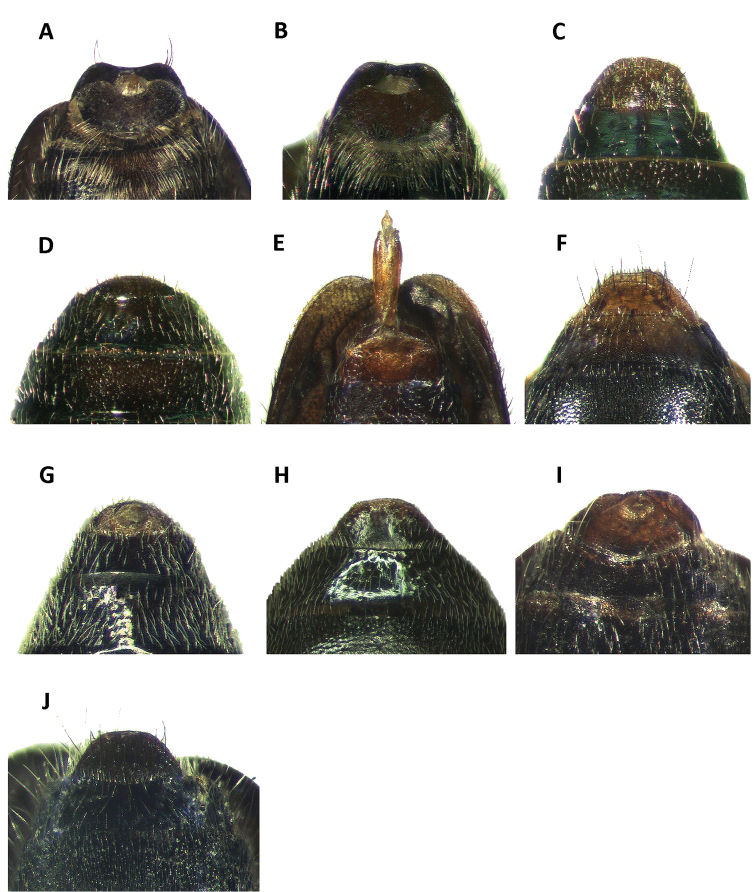
Pygidium of: **A** *Neocallotillus elegans* (*Callotillus elegans elegans*) male **B** *Neocallotillus elegans* (*Callotillus elegans vafer*) male **C** *Neocallotillus elegans* (*Callotillus elegans elegans*) female **D** *Neocallotillus elegans* (*Callotillus elegans vafer*) female **E** *Neocallotillus intricatus* (*Callotillus intricatus*) male **F** *Neocallotillus intricatus* (*Callotillus intricatus*) female **G** *Callotillus bahamensis* male **H** *Callotillus bahamensis* female **I** *Callotillus eburneocinctus* male **J** *Callotillus eburneocinctus* female.

Abdomen: Smooth to glossy, moderately vested, feebly to moderately convex, with six visible ventrites; lateral margins of ventrites 1-5 parallel, posterior margins truncate; sixth ventrite triangular to subquadrate in shape; male pygidia moderately differentiated from female pygidia (Fig. 5A–F).

Aedeagus: Moderately robust; phallobasic apodeme short, slender distally; endophallic struts elongate, slender throughout their length.

Etymology: This generic name, which is preceded by the Latin prefix *neo* (new), refers to the superficial similarity to the genus *Callotillus*.

##### Remarks.

Expressing tentative assignment of some of his species to *Callotillus*, Wolcott (1923) wrote: “*Callotillus crusoe*, as well as *Callotillus elegans* and *Callotillus vafer*, are placed in *Callotillus* provisionally only, as it differs from the other members of the genus in several important characters. No doubt, eventually, the creation of a new genus will be necessary for the reception of this new species and *Callotillus elegans* and *Callotillus vafer*. In *Callotillus eburneocinctus*, the terminal segment of the maxillary palps is sub-cylindrical, the eyes are emarginate internally and the abdomen has but five segments. In *Callotillus elegans*, *Callotillus vafer*, and *Callotillus crusoe* the maxillary palpi have the terminal segment conical, the eyes are deeply emarginate anteriorly, and the abdomen has six distinct well developed segments”. The morphological differences listed by Wolcott, together with the presence of pectinate antennae on males of *Neocallotillus* versus serrate antennae on males of *Callotillus* (Fig. 3A–C, G–H), and an elytral swelling present in *Callotillus* but absent in *Neocallotillus* (Fig. 4 A–B), support the recognition of two genera within the group. The monotypic *Barrotillus* was also examined in this study in order to assess possible congenericity with *Neocallotillus*. The structure of the antennae and number of antennomeres serve as evidence to conclude that these closely related genera should be considered as separate taxa (Fig. 3A–K).

#### Neocallotillus elegans

Taxon classificationAnimaliaColeopteraCleridae

(Erichson, 1847) comb. n.

Tillus elegans Erichson 1847: 85.
Callotillus occidentallis Gorham 1882: 129.
Callotillus vafer Wolcott 1921: 270 **syn. n.**

##### Holotype depository.


 Zoologisches Museum Berlin, Germany (ZMB). Holotype locality: “Republica Peruana”.

##### Distribution.

USA: AZ, CA, LA, NM, NV, TX, UT; Mexico: Baja California, Baja California Sur, Chiapas, Chihuahua, Guerrero, Jalisco, Morelos, Nayarit, Oaxaca, Sonora, Tamaulipas, Yucatan; Central America: Costa Rica, Guatemala, Honduras, Nicaragua (Fig. 8A).

##### Differential diagnosis.

*Neocallotillus elegans* can be differentiated from similar species based on the integument color, fascia pattern, and wide geographic distribution. The species is most similar to *Neocallotillus intricatus* but can be easily differentiated from the latter based on the fasciae pattern on the elytral disc. *Neocallotillus elegans* has the elytra adorned with a light testaceous to almost whitish median, longitudinal, slightly protruding fascia, and a pair of protruding maculae on the anterior half near the humeral angles (Fig. 1A–C), these maculae may be absent in some individuals (Fig. 1D). *Neocallotillus intricatus* has the elytral disc decorated with an intricate design of light testaceous fasciae and a pair maculae arranged in the following manner: each elytron with one macula situated posterior to the humeral angle; one strongly procurved fasciae located on the anterior half of the elytral disc, this fascia initiates on the elytral suture and do not reach the epipleural fold; and a second fascia situated immediately posterior to the first, this band is strongly oblique, initiating on the epipleural fold and not reaching the elytral suture (Fig. 1E). The geographic distribution of these species can also serve to separate them. *Neocallotillus elegans* is found from the United States to Costa Rica (Fig. 8A), while *Neocallotillus intricatus* is restricted to Costa Rica and Panama (Fig. 8B).

##### Redescription.

Form: Small individuals, feebly to moderately slender (Fig. 1A–D). Body: elongate, slender. Color: body integument variously colored, from piceous to ferruginous, with tones ranging from fuscous to testaceous; each elytron with one macula and one fascia, both markings ranging from almost albus to testaceous; thefascia is located on the median region of the elytral disc and can range from conspicuously wide to almost imperceptible; the macula is located on the median region of the first third of the elytral disc, initiating on the epipleural fold and almost reaching the elytral suture; these markings can be medially interconnected or not. The maculae may be absent in some specimens (Fig. 1D).

**Figure 6.**
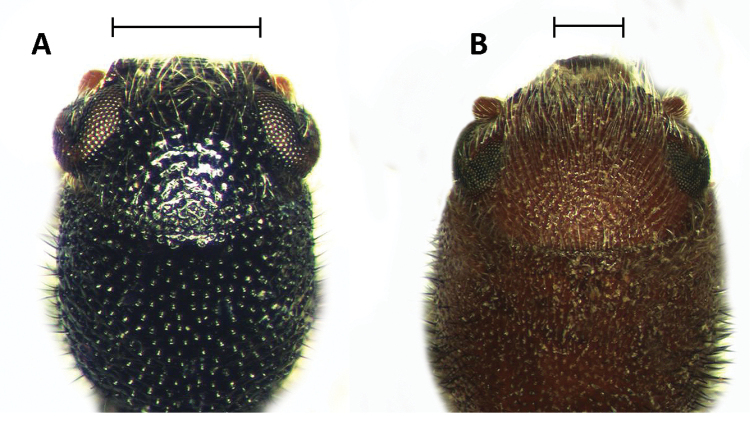
Head of: **A** *Neocallotillus elegans* (*Callotillus elegans elegans*) **B** *Callotillus eburneocinctus*. Scale bars indicate width of frons.

Head: Including eyes wider than pronotum; eyes conspicuously bulging laterally, taller than wide, large, finely faceted, very strongly emarginate; emargination subtriangular, extending 3/4 the eye width; integument moderately to strongly punctate; antennal notch located in front of antennal emargination; frons feebly to moderately bi-impressed. Antennae consisting of 10 antennomeres; antennomeres 2-3 small, beadlike; fourth antennomere strongly serrate, robust; antennomeres 4-9 pectinate, gradually increasing in size toward distal end; last antennomere enlarged, ovoid in shape, laterally compressed (Fig. 3A, B). Anterior portion of clypeus wide, approximately 3× the length of eye emargination (Fig. 6A).

Thorax: Pronotum longer than broad, moderately to strongly punctate, punctations ranging from coarse and deep to moderately shallow and fine; sides subparallel in dorsal view, then abruptly constricted on posterior fourth; disc feebly convex. Prosternum smooth to feebly punctate; punctations coarse, finely to moderately vested with pale, recumbent setae. Mesoventrite smooth, feebly punctate; finely vested with some pale, semi-recumbent to recumbent setae. Metaventrite moderately punctate; strongly convex; surface smooth, vested with fine, recumbent and semi-recumbent setae; longitudinal depression present; metaventral process absent.

Elytra: Humeri indicated, slender, elongate; lateral margins subparallel, slightly to moderately broader on last third, then moderately to strongly depressed on second third, and conspicuously convex again on last third; sculpture consisting on shallow, irregularly arranged punctations; elytral apices subtriangular to almost rounded, feebly dehiscent; interstices at elytral base about 3× the width of punctuation; scutellum subquadrate, not depressed; epipleural fold complete, narrowing toward apex.

Legs: Femora swollen on posterior half; shiny; very feebly rugulose; weakly clothed with some semi-recumbent setae. Tibiae more profusely vested than femora.

Abdomen: Six ventrites; ventrites 1-5 shiny, smooth, subquadrate, not depressed laterally. Fifth ventrite subquadrate; lateral margins subparallel; posterior margin broadly, shallowly emarginate. Sixth ventrite small, conspicuously excavated, moderately, coarsely punctate, conspicuously broader than long; lateral margins strongly oblique, procurved; posterior margin broadly, moderately deeply, U-shaped emarginate; posterolateral angles broadly rounded (Fig. 5A–B). Fifth tergite subquadrate, moderately, coarsely punctate; posterior margin broadly, shallowly emarginate. Sixth tergite concave, wider than long; surface smooth; lateral margins moderately oblique; posterior margin broadly, moderately deeply, U-shaped emarginate. Posterolateral angles broadly rounded, fully covering sixth ventrite from dorsal view.

**Figure 7.**
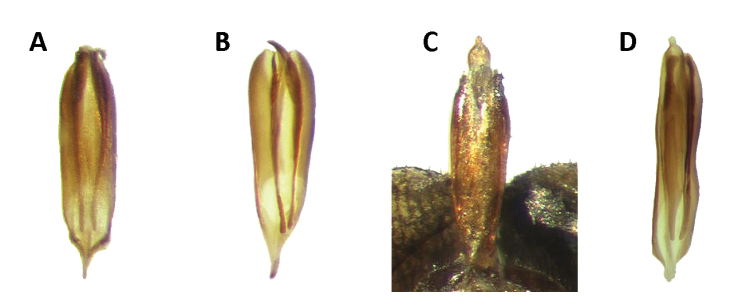
Aedeagus of: **A** *Neocallotillus elegans* (*Callotillus elegans elegans*) **B** *Neocallotillus elegans* (*Callotillus elegans vafer*) **C** *Neocallotillus intricatus* (*Callotillus intricatus*) **D** *Callotillus eburneocinctus*.

Aedeagus: Phallobasic apodeme present; phallus with copulatory piece moderately swollen at apex; phallic plate devoid of denticles; intraspicular plate present, elongate; phallobasic apodeme short, expanded distally; phallobase subparallel; phallobasic lobes free; tegmen complete, fully covering phallus; phallobasic lobes pointed anteriorly; endophallic struts long, extending beyond the length of tegmen; endophallic struts slender throughout their length, weakly robust distally (Fig. 7A–B).

Female variation: Females can be distinguished from males based on the antennal structure and the shape of the last abdominal segment. The antennal shape of females is moderately to strongly serrate; antennomeres 2-3 are slender, filiform; antennomeres 4-9 are serrate, the serrations gradually increase in size toward distal end (Fig. 3D, E). The posterior margin of the sixth ventrite of females is strongly procurved, producing a semicircular pygidium (Fig. 5C–D).

##### Remarks.

**Figure 8.**
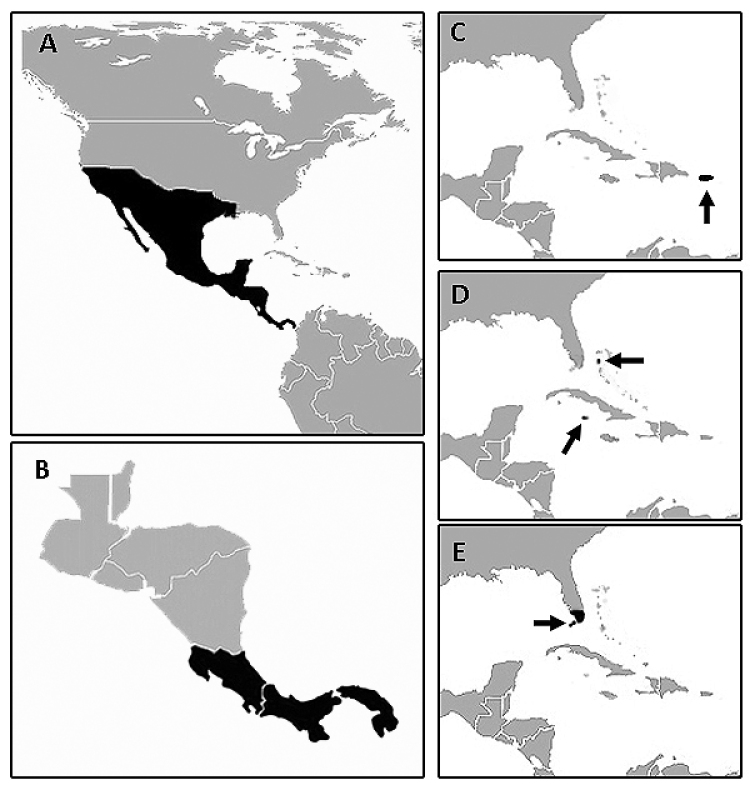
Distribution map of: **A** *Neocallotillus elegans* **B** *Neocallotillus intricatus* **C** *Neocallotillus crusoe* **D** *Callotillus bahamensis* **E** *Callotillus eburneocinctus*.

The species *Callotillus occidentalis*, described by Gorham (1882) for individuals collected in Guatemala and Nicaragua, was later synonymized with *Callotillus elegans* by Schenkling (1903). We examined one female paratype of *Callotillus occidentallis* (Fig. 2A) and agree with Schenkling’s synonomy. Barr (1950) proposed that *Callotillus vafer* be reclassified as a subspecies of *Callotillus elegans* on the basis of integument color, geographic distribution discontinuity, and differences in the structure of the elytral punctation. Individuals inhabiting Arizona, California, Nevada, New Mexico, western Texas, Utah, and the Baja California peninsula were classified by Barr as *Callotillus elegans vafer* while specimens from western Louisiana, eastern Texas, Mexico and Central America were recognized as *Callotillus elegans elegans*. Barr also indicated the existence of intermediate forms of these subspecies in the United States and Baja California; however, we have found intermediate forms exist throughout much of the geographic range of the species. It is possible to find both color morphotypes (Fig. 1A–B), including intermediate forms (Fig. 1C–D), as well as conspicuously similar antennal forms (Fig. 3A, B), throughout North and Central America. Aside from integument variation, no other morphological evidence was found to differentiate these subspecies as separate taxa. As more material from these subspecies has been accumulated, we consider *Neocallotillus elegans* a species with a wide spectrum of color variation throughout an extensive geographic distribution (Fig. 8A).

##### Material examined.

PARATYPE: 1 female: [*Callotillus occidentalis* Gorham], Pantaleon, 1700 ft., Champion, paratype depository: BMNH. (Fig. 2A).

##### Additional material examined

(N= 241). USA: 2 males: SW Hidalgo Co., TX, 17-III-1946, George B. Vogt, beating flowers and foliage, on *Prosonis juliflora* DeCandolie; 1 male, 3 females: Riverside Co., CA, Chuckawalla Mts., Corn Spg. Campground, 25-IV-1987, A. J. Mayor; 1 female: Imperial Co., CA, 9 mi N Winterhaven, 2-IV-1997, F. G. Andrews and A. J. Gilbert, sweeping *Prosopis*; 1 female: TX, 12 mi W Guthrie, 14-VII-1969, K. Polk; 1 male, 1 female: Hidalgo Co., TX, Sta. Ana Natl. Refuge, vic. Willow Lake, T. C. McRea; 1 male, 1 female: Val Verde Co., TX., Pecos River, 29-VIII-1970, no collector data; 1 male: Rio Grande City, Starr Co., TX, on *Prosopis*; 1 male: NM, 12 mi W Carlsbad, 25-IV-1971, on mesquite, C. R. Ward; 1 male: San Diego Co., CA, Borrego State Park, 17-20-IV-1969, no collector data; 1 female: Painted Canyon, Riverside Co., Calif., 25-III-1962, F. G. Andrews; 1 male: N. M., Hidalgo Co., Coronado Natl. Forest, 26-V-1976, W. Iselin; 3 males, 2 females: AZ, Sta. Catalina Mts., Pima Canyon, 7-IX-1970, K. Stephan; 1 female: TX, 5 mi NW of Alpine, 17-VI-1965, on *Sapindus drummondii*, G. H. Nelson; 2 males: Starr Co., TX, 2 mi W of Sullivan City, reared from *Pithecelobium flexicaule*, G. H. Nelson; 1 female: Socorro Co., NM, Bosque de Apache Nat. Wildlife Ref., 2-VII-2000, F. W. Skillman Jr.; 2 females: AZ, Sta. Catalina Mts., Pima Canyon, Bred ex Palo Verde, K. Stephan; 1 female AZ, Sta. Catalina Mts., Sabino Canyon, 11-VIII-1961, G. H. Nelson;; 1 female: CA, Imperial Co., 7 mi N of Glamis, 29-VIII-1987, on *Cercidium floridum*, Wood; 2 males, 1 female: Imperial Co., CA, Frink Spr., 7-VII-1993, on *Olneya tesota*, G. H. Nelson; 1 female: San Diego Co., CA, 3 mi E of Jacumba, reared *Acacia greggii*, 22-V-1987, G. H. Nelson; 1 male, 3 females: AZ, Pima Co., Green Valley, 15-VII-976, no collector data; 1 male, 1 female: Eddy Co., NM, 26 mi E of Carlsbad, 2-VI-1977, no collector data; 1 female: Dona Ana Co., NM, 9 miles west of Santa Teresa, 8-V-1999, J. C. Schaffner; 1 female: Bastrop Co., TX, Bastrop, 3-VI-1997, S. G. Wellso; 1 female: Cochise Co., AZ, 12 mi N of Douglas, 24-VII-1982, J. E. Wappes; 1 male: TX, 3 mi southeast Presidio, 12-IV-1968, J. G. Hafernik; 1 male, 1 female: Pima Co., AZ, Mt. Lemon, V-17-1976, R. Lenczy; 1 male: Cochise Co., AZ, Wilcox Dry Lake, 6-VI-1970, A. R. Hardy; 1 male: TX, Brownsville, VII-1937, H. S. Barber; 2 males: AZ, Tucson, VIII-193 [], Bryant; 1 female: Riverside Co., CA, 25-III-1962, F. G. Andrews; 10 males, 5 females: CA, Mecca, 20-V-1924, B. Warwick; 1 female: CA, Calipatria, 6-V-1924, B. Warwick; 1 female: CA, Calipatria, 1-V-1924, B. Warwick; 1 male: CA, Calipatria, 10-V-1924, B. Warwick; 10 females: Hidalgo Co., TX, J. N. Knull, 28-III-1954; 3 males, 4 females: TX, Brownsville, , 25-V-1934, J. N. Knull; 2 females: Cameron Co., TX, 25-III-1952, J. N. Knull; 5 males, 3 females: Hidalgo Co., TX, 26-III-1957, J. N. Knull; 6 males, 6 females: Hidalgo Co., TX, 20-III-1952, D. J. and J. N Knull; 1 male: AZ, Huachuca Mt., 5-VI D. J. and J. N. Knull; 2 females: Starr Co., TX, D. J. and J. N. Knull, 28-III-1950; 1 male: Uvalde Co., TX, 20-V, D. J. Knull; 1 male, 2 females: Hidalgo Co., TX, 29-III-1968, D. J. and J. N Knull; 2 females: Hidalgo Co., TX, 26-III-1953, D. J. and J. N. Knull; 1 male, 1 female: Hidalgo Co., TX, 24-III-1954, D. J. and J. N. Knull; 1 male: Hidalgo Co., TX, 28-III-1954, D. J. and J. N. Knull; 1 male, 4 females: CA, Santa Rosa L., VIII, J. L. Knull; 3 males, 1 female: AZ, Wilcox, 4-VII-1951, D. J. and J. N. Knull; 2 males: AZ, Wilcox, 6-VI-1954, D. J. and J. N. Knull; 1 female: AZ, Patagonia Mts., 2-VII-1953, D. J. and J. N. Knull; 1 male: Culberson Co., TX, 9-VII-1953, D. J. and J. N. Knull; 2 male, 2 females: Pima Co., AZ, 9-VII-1975, N. M. Downy; 1 male: Bell Co., TX, Holland, 12-VII-1988, S. G. Wellso; 2 males: Hidalgo Co. TX, Sta. Ana Natl. Refugee, VIII-1977, J. E. Wappes; 1 male, 1 female: Calipatria Co., CA, 15-VII-1925, B. Warwick; 1 female: Uvalde Co., TX, VII-27, J. N. Knull; 1 female: CA, Mecca, 12-V-1924, B. Warwick; 4 males, 1 female: CA, Calipatria, 1-6-V-1924, B. Warwick; 15 males, 9 females: AZ, Chiricahua Mts., 1-3-VI, J. N. Knull; 1 male, 2 females: AZ, Tucson, VIII-19, J. N. Knull; 2 females: Hidalgo Co., NM, 24-III-1954; D. J. and J. N. Knull; AZ, Wilcox, 11-VI-1954, D. J. and J. N. Knull; 2 males: Imperial Co., CA, 15 mi W of Calexico, 5-6-VI-1961, light trap, H. F. Howden; 1 female: CA, Palm Springs, 15-VI-1948, D. J. and J. N. Knull; 1 female: TX, Davis Mts., 24-VI-1957, D. J. and J. N. Knull; 4 males, 7 females: TX, Chisos Mts., V-25, J. N. Knull; 2 males: Jeff Davis Co., TX, 20-VI-1957, D. J. and J. N. Knull; 1 male: TX, on live oak, 17-V-1965, J. L. Bottmer; 2 males: AZ, Mt. Huachuca, 5-8-VI, D. J. and J. N. Knull; 3 males: Hidalgo Co., TX, 20-IV-1968, D. J. and J. N Knull; 4 males, 6 females: Jim Wells Co., TX, 8 mi S of Alice, 6-8-April-1984, S. G. Wellso; 2 females: Jim Wells Co., TX, Alice, 15-IV-1986, S. G. Wellso; 1 male: Brewster Co., TX, Castolon, 14-IV-1983, S. G. Wellso; 1 female: Brewster Co., TX, Big Bend Natl. Park, 16-IV-1983, S. G. Wellso; MEXICO: 1 male, 1 female: Chiapas, Mex., 4 mi NW of Pueblo Nuevo River Bajada, 15-VII-1965, G. H. Nelson; 1 male: Baja Calif. S., Mex., 4 mi S La Paz, 14-IX-1978, B. K. Dozier; 1 male: Baja Calif., Mex., Catavina, riparian palm oasis, on *Acacia greggii*, G. H. Nelson; 1 male, 2 females: Baja Calif. S., Mex., La Paz, 29-VI-1973, B. F. Chamberlain; 2 females: Baja Calif. S., Mex., 1-3 mi E Cabo San Lucas, G. Riley; 1 female: Baja Calif. Sur, Mex, 9 mi N San Jose del Cabo, G. Riley; 2 males: Baja Calif. S., Mex., 66 km NE Insurgentes nr. Ultima Agua, on *Prosopis articulata*, 13-IV-1994, D. Yanega; 2 males: Morelos, Mex., Tlaquiltenango, Huaxtla, 18.37598 N, 99.04804 W, 1053 m, 13-XII-2009, V. H. Toledo; 1 male: Sonora, Mex., 29 km SE Tecoripa y 3 km S Rancho Las Peñitas, 733 m, on *Acacia* sp., 22-IV-2004, V. H. Toledo; 2 males, 1 female: Baja Calif. S., Mex., Las Barrancas, 27-V-1984, P. DeBach, Malaise trap; 1 female: Yucatan, Mex., Tekom, 04-VIII-1940, I. Sanderson; 1 male: Baja Calif., Mex., Santa Rosa, 08-10-I-1914, G. Beyer; 1 male, 2 females: Chiapas, Mex., 4 mi NW of Pueblo Nuevo, 15-VII-1968, G. H. Nelson; 1 male: Tamaulipas, Mex., El Encino, 15-IV-1984, S. G. Wellso. 8 males, 3 females: Baja Calif. S., Mex. 6 km E of San Antonio, 350 m, on *Prosopis articulata*, 11-IV-1994, no collector data; CENTRAL AMERICA: 2 males: Guanacaste, [Costa Rica], Cerro El Hacha, 800m, 12 km SE La Cruz, 320000, 364000, 1998; 1 male: Heredia Province, Costa Rica, Sarapiqui, Chilamate, La Marita Farm, 26-II-1992, R. L. Johnson and R. Ochoa; 1 female: Rivas, Nicaragua, San Juan del Sur, 11’ 15° N, 82’ 52° W, 10-III-1998, L. J. Clark; 1 female: Granada, Nicaragua, Volcan Mombacho, Finca San Joaquin, 15-V-1998, malaise trap, in organic coffee, J. M. Maes.

#### Neocallotillus crusoe

Taxon classificationAnimaliaColeopteraCleridae

(Wolcott, 1921) comb. n.

##### Holotype depository.

(AMNH). Holotype locality: Camuy, Puerto Rico.

##### Distribution.

Puerto Rico (Fig. 8C).

##### Differential diagnosis.

*Neocallotillus crusoe* is similar to *Neocallotillus elegans* but differs from the latter species by the absence of seriate elytral punctures, its larger size, its broader form, and the impunctate metaventrite and abdomen. The differently formed and arranged raised fasciae or maculae are also distinguishing characters. The head and pronotum in *Neocallotillus crusoe* are densely pubescent, sparsely so in the *Neocallotillus elegans* species; the antennae are differently formed, having a greater number of triangular segments; the color pattern is unique; the arrangement of the pubescence in basal half of elytra is distinctive; and the densely pilose elytral tubercles are present only in *Callotillus eburneocinctus*.

##### The following is Wolcott’s original description.

N=1. Form: Moderately slender. Color: Black. Dorsal surface rather feebly shining; ventral surface very shining; front of head narrowly rugulose; antenna (apical two segments black) and labrum at sides testaceous; elytra black with the apical half in large part pale yellow; a large, ovate, ante-apical, black maculation; sides at middle with an oblique, elevated, white maculation; a similar minute, slightly transverse maculation at basal fourth at middle of width of each elytron.

Head: Including the not prominent eyes, equal in width to pronotum at apex; surface rather coarsely rugoso-punctate; pubescence dense, semi-recumbent, grayish white. Antennae slightly longer than head and prothorax, ten-segmented; basal segment short, very stout; second small, subtriangular; third to ninth triangular, their apices acute; ninth and tenth forming an elongate ovate mass; tenth narrower than eighth, nearly as long as seventh and eighth together; color testaceous; ninth and tenth segments black, the former narrowly testaceous at base.

Pronotum: Slightly longer than wide; apical margin truncate; sides parallel to slightly behind the middle, then rather strongly arcuately narrowing to about basal fourth, thence subparallel to base; base truncate, the extreme edge with a fine elevated margin; subapical constriction wanting; subapical transverse impression nearly obsolete, only faintly indicated in certain lights; surface with sculpture same as that of head; pubescence same as that of head but with long, sparse, erect, black hairs intermixed.

Elytra: Base nearly twice as wide as pronotum at base; length 1/3× times width at base; humeri obtusely rounded; sides from humeri to middle straight, nearly parallel, behind the middle gradually broadening to apical fourth, thence arcuately narrowing to the conjointly rounded apices; color black, apical half pale yellow, anterior margin of yellow portion convex; in apical third a large, elongate ovate, common, sutural maculation, extending very nearly to apical margin, black; sides slightly anterior to middle with a feebly arcuate, linear, elevated, white maculation, this extending obliquely and attenuate forward from lateral margin halfway to suture; at basal fourth a minute, slightly transverse, elevated, white maculation midway between the lateral margin and the suture; base with a broad triangular area, having one angle on suture, and an oblique fascia each side, extending from immediately behind the humeri to the suture at a point slightly before the middle, composed of dense, coarse, grayish-white pubescence; a large, feebly elevated, subbasal tubercle, midway between lateral margin and suture, densely clothed with a tuft of long, black hairs; black portions densely clothed with short, semi-recumbent, black pubescence, longer and erect in humeral region; the yellow portion densely clothed with pale yellowish pubescence, a few nearly erect, long, black hairs intermixed; surface finely and sparsely punctate at extreme base, becoming closer at about basal fourth, and a little coarser toward the apex; punctuation irregular throughout, showing no tendency to become seriate.

Abdomen: Impunctate; very sparsely clothed with long, black hairs. Meso[ventrite]sternum smooth; moderately clothed with semi-recumbent, grayish-white pubescence. Legs rather short and stout; moderately clothed with rather long, white hairs. Length, 4.2 mm.

##### Remarks.

Wolcott (1923) described *Callotillus crusoe* from a single male specimen collected near Camuy, Puerto Rico. Wolcott concluded that this species was allied to *Callotillus elegans* and *Callotillus vafer* but could be differentiated from the latter two based on the absence of elytral punctations, a relatively larger size and broader body shape, and the absences of punctations on the metaventrite and abdomen. Based on Wolcott’s illustration and his descriptive work, the shape of the antennae appear to be serrate, and the species seems to be comparatively larger and broader than remaining *Neocallotillus* species. These characteristics may suggest a relationship to *Callotillus*. Due to the absence of material of *Neocallotillus crusoe*, a redescription of this species is not presented in this study; however, in order to complement the revision of the newly erected genus *Neocallotillus*, the descriptive work given by Wolcott (1923) is transcribed above. Based on Wolcott’s assessment, we tentatively place *Callotillus crusoe* within *Neocallotillus*. Further examination of material from this species will serve to corroborate the relatedness of this species with those species composing *Neocallotillus*, or conversely, its reassignment to *Callotillus*.

#### Neocallotillus intricatus

Taxon classificationAnimaliaColeopteraCleridae

(Wolcott & Dybas, 1947) comb. n.

##### Holotype depository.


Naturalis Biodiversity Center, Leiden, The Netherlands (RMNH). Holotype locality: Farm La Caja, 8 km. west of San Jose, Costa Rica.

##### Distribution.

Costa Rica, Panama (Fig. 8B).

##### Differential diagnosis.

*Neocallotillus intricatus* is most similar to *Neocallotillus elegans*. The two species can be differentiated based on the fasciae pattern on the elytra disc. *Neocallotillus intricatus* has the elytral disc decorated with an intricate design of light testaceous fasciae and a pair of maculae arranged in the following manner: Each elytron with one macula situated posterior to the humeral angle; one strongly procurved fasciae located on the anterior half of the elytral disc, this fascia initiates on the elytral suture and do not reach the epipleural fold; and a second fascia situated immediately posterior to the first, this band is strongly oblique, initiating on the epipleural fold and not reaching the elytral suture (Fig. 1E). *Neocallotillus elegans* has the elytra adorned with a light testaceous to almost whitish median, longitudinal, slightly protruding fascia, and a pair of protruding maculae on the anterior half near the humeral angles (Fig. 1A–C), these maculae may be absent in some individuals (Fig. 1D). The geographic distribution of these species can also serve to separate them. *Neocallotillus intricatus* is limited to Costa Rica and Panama (Fig. 8B) while *Neocallotillus elegans* is found from the United States to Costa Rica (Fig. 8A).

##### Redescription.

Form: Body elongate; head, pronotum and anterior half of elytra slender, feebly expanded behind second half of elytral margins. Color: Head, pronotum, thorax, abdominal segments 1-4 and femora griscent to fuscous; anterior margin of pronotum, antennae, mouthparts, tibiae, abdominal segments 5-6 and elytral apex light-ferruginous. Elytra adorned with an intricate array of two pale-testaceous fasciae and a pair of maculae of the same color, the position of these elytral markings is as follows: the first fascia is located on the anterior half of the elytral disc, this band is strongly procurved, initiating on the elytral suture and not reaching the epipleural fold; the second fascia is located immediately posterior to the first band and is moderately oblique, initiating on the epipleural fold and not reaching the elytral suture; the two macuale are located posterior to the humeral angles. Elytral pattern not elevated from elytral disc (Fig. 1E).

Head: Including eyes not wider than pronotum; eyes strongly emarginate, taller than wide, feebly bulging laterally, rather small, finely faceted; emargination subtriangular; integument moderately punctate; antennal notch anterior to antennal emargination; frons moderately bi-impressed. Antennae of male consisting of 10 antennomeres; antennomeres 2-3 small, beadlike; fourth antennomere serrate; antennomeres 4-9 pectinate, gradually increasing in size toward distal portion of antenna; last antennomer enlarged, as long as ninth antennomere, ovoid in shape, laterally compressed.

Thorax: Pronotum longer than broad; surface rugulose and strongly, finely punctate; punctations numerous, shallow; sides subparallel in dorsal view, then abruptly constricted on posterior fourth; disc convex. Prosternum feebly convex; surface smooth; conspicuously punctate, punctations shallow. Mesoventrite smooth; surface feebly punctate; finely vested. Metaventrite globate; surface smooth, strongly convex and finely punctate; longitudinal depression and metaventral process absent; metepisternum exposed but profusely covered with short, fine, pale setae observable in lateral view. Scutellum ovoid in shape.

Elytra: Humeri indicated; slender on anterior half and then gradually expanding behind middle; surface convex on first third, then strongly depressed on second third, and then conspicuously convex on last third; elytral sinuosity observable in lateral view; sculpture on elytral disc consisting on abundant, very shallow, irregularly arranged punctations almost imperceptible in some individuals; elytral apices rounded, moderately dehiscent; interstices on elytral base about 2× the width of punctuation; epipleural fold complete, narrowing toward apex.

Legs: Femora swollen; surface shiny, smooth; vestiture consisting of some semi-recumbent setae, then abruptly vested with numerous pale, semi-recumbent, rather stout setae on distal face. Tibiae more profusely vested than femora; vestiture consisting on stout, pale, short, recumbent setae interspaced with some semierect setae.

Abdomen: Six ventrites; ventrites 1-4 broadly convex, smooth, rugulose, subquadrate, not depressed laterally; posterior margins truncate. Fifth ventrite shiny; lateral margins moderately obtuse; posterior margin broadly, shallowly emarginate. Sixth ventrite small; surface moderately excavated, shiny, feebly punctate, conspicuously broader than long; lateral and posterior margins strongly oblique, nearly semicircularly rounded (Fig. 5E). Fifth tergite sub-quadrate; surface moderately, coarsely punctate; posterior margin broadly, shallowly emarginate. Sixth tergite feebly concave, wider than long; surface smooth; lateral margins moderately oblique; posterior margin truncate; posterolateral angles subquadrate. Sixth tergite extending beyond apical margin of sixth ventrite, fully covering sixth ventrite from dorsal view.

Aedeagus: Moderately robust; distal portion of phallus petiolate; phallobasic apodeme present; phallus with copulatory piece moderately swollen distally; intraspicular plate present, elongate; phallobasic lobes moderately procurved; tegmen complete, fully covering phallus; phallobasic lobes acuminate distally (Fig. 7C).

Female variation: The antennal shape of females is strongly serrate (Fig. 3F), rather than pectinate, as observed in males (Fig. 3C). Females have antennomeres 1-3 slender, antennomeres 4-5 are moderately serrate, and antennomeres 6-9 are strongly serrate, serrations gradually increase in size toward the distal end. Females also have the lateral and posterior margins of the sixth ventrite subquadrate (Fig. 5F), producing a somewhat semicircular pygidium. All females in the material examined were moderately larger than males.

##### Remarks.

Wolcott and Dybas (1947) described *Callotillus intricatus* based on two specimens, one male and one female (Fig. 2B), collected from a single locality 8 km west of San Jose, Costa Rica. This species is here transferred to *Neocallotillus* based on the pectinate antennae of male individuals (Fig. 3C) and the feebly pectinate to almost serrate antennae in females (Fig. 3F), these antennal shapes are very similar to those observed on *Neocallotillus elegans* (Fig. 3A–B, D–E). Other characters that are similar in these species are general body shape (Fig. 1A–E), elytral sculpturing, and the conical shape of the terminal segment of the maxillary palps.

##### Material examined.

PARATYPE: 1 male: Farm La Caja, 8 km W San José, Costa Rica, Eing, 12-VI to 20-VII-1924, hand written red label paratype depository: CNHM.

##### Additional material examined (N=9).

2 females: Costa Rica, Guanacaste, 3 km SW of R. Naranjo, 11-18-III-1992, F. D. Parker; 1 male, 3 females: Costa Rica, Guanacaste, 14 km S Cañas, 2-III-1990, F. D. Parker; 1 male, 2 females: Panama, Coclé province, El Valle [Anton], 19-II-1999, W. E. Wappes.

#### Callotillus

Taxon classificationAnimaliaColeopteraCleridae

Wolcott, 1911

##### Type species.

*Callotillus eburneocinctus* Wolcott, 1911 (original designation).

##### Differential diagnosis.

*Callotillus* is most similar to the new genus *Neocallotillus*. A list of useful characters to properly differentiate these genera is provided above in the differential diagnosis of *Neocallotillus*.

##### Redescription.

Size: 6–10 mm. Color: Testaceous and ferrugineous to almost black; fasciae on elytral disc may be present or not, if present, ranging from light testaceous to brown. Body moderately robust, expanded posteriorly.

Head: Rather small, longer than wide; eyes inconspicuously bulging laterally (Fig. 6B), strongly emarginate at antennal insertion; clypeus feebly emarginate medially; antennae composed of 10 antennomeres; sexual dimorphism observable in antennal composition, with antennomeres 2-9 in males conspicuously serrate (Fig. 3G, H) but moderately serrate in females (Fig. 3I, J), last antennomere compressed laterally in both sexes; labrum moderately constricted laterally, subquadrate; terminal maxillary palps sub-cylindrical; terminal labial palps securiform.

Thorax: Pronotum moderately to conspicuously globose, narrower than anterior margin of elytra; disc moderately to strongly convex, inconspicuously broader at middle, feebly sinuate behind middle, then feebly to moderately constricted on last fourth; anterior depression and antescutellar impression absent. Prosternum: smooth to feebly puncticulate; conspicuously wider than long. Mesoventrite smooth, feebly to moderately puncticulate; metepisternum partially visible in lateral view, not fully concealed by elytron. Metaventrite moderately to strongly convex; surface variously punctate.

Elytra: Moderately robust, elongate, expanded posteriorly; median region of elytral disc feebly to moderately depressed in lateral view; subbasal elytral swellings present; elytral apex declivity feebly to moderately steep; transverse fasciae may be present on elytral disc or not, if present elevated from elytral disc.

Legs: Femora wide; surface rugulose to smooth. Tibiae rugulose, moderately expanded posteriorly; tibial spur formula 2-2-2. Two tarsal denticles, inner tarsal denticles trigonal, outer tarsal denticles digitiform.

Abdomen: Six ventrites; surface smooth, moderately convex; ventrites 1-5 with lateral margins parallel and posterior margins truncate; male pygidium moderately differentiated form that of females.

Aedeagus: Slender and moderately sclerotized; phallobasic apodeme robust distally; endophallic struts short, robust throughout their length.

##### Remarks.

In accordance to the taxonomic changes previously discussed, in this revision we treat *Callotillus* as a genus containing two species: *Callotillus bahamensis* Vaurie (Figs 1G, 2C), a species currently recorded from the Bahamas and the Cayman Islands (Fig. 8D), and *Callotillus eburneocinctus* Wolcott (Figs 1H, 4B), a species restricted to the southernmost tip of the Florida peninsula, including the Florida Keys (Fig. 8E).

#### Callotillus bahamensis

Taxon classificationAnimaliaColeopteraCleridae

Vaurie, 1952

##### Holotype depository.


American Museum of Natural History
(AMNH). Holotype locality: South Bimini Island, Bahamas, British West Indies.

##### Distribution.

The Bahamas, Cayman Islands (Fig. 8D).

##### Differential diagnosis.

*Callotillus bahamensis* is most similar to *Callotillus eburneocinctus*. The two species can be differentiated with ease based on the color pattern on the elytral disc. *Callotillus bahamensis* has the elytral disc predominantly piceous, except a light testaceous area surrounding the humeral angles, this testaceous area extends from the anterior fourth of the epipleural fold and may reach the scutellum or not (Fig. 1G). *Callotillus eburneocinctus* has the anterior half of the elytral disc rufous and the posterior portion fuscous, this coloration shift is interrupted by a transverse, moderately elevated, pale band which runs from the elytral suture to the epipleural fold (Fig. 1H).

##### Redescription.

Form: Moderately robust; elytra gradually expanded toward apex, then abruptly narrowing behind distal fourth. Color: Anterior portion of femora, trochanters, coxae, and anterior fourth of the elytral disc light testaceous, this testaceous pattern on the elytral disc reaches the humeral region laterally and the scutellum internally; remaining body uniformly fuscous; the elytral disc is devoid of any bands or fasciae (Fig. 1G).

Head: Including eyes not wider than pronotum; eyes taller than wide, not bulging laterally, rather small, finely faceted, strongly sub-triangularly emarginate; integument rugose, feebly punctate, punctuation rather small; antennal notch located in front of emargination; frons not bi-impressed. Antennae of males composed of 10 antennomeres; second antennomere short, robust, beadlike in shape; third antennomere about 2× the length of previous antennomere, moderately serrate; fourth antennomeres slightly longer than third antennomere; antennomeres 4-9 about the same length, strongly serrate; last antennomere elongate, about 2.5× the length of ninth antennomere, slightly ovoid in shape, laterally compressed (Fig. 3G).

Thorax: Pronotum globose, slightly broader than long; surface shiny, finely, deeply punctate; sides subparallel, then abruptly constricted on posterior fourth; disc strongly convex; anterior transverse depression and subbasal tumescence absent. Prosternum wider than long; surface smooth. Mesoventrite rugulose; surface finely punctate, feebly vested with fine, pale, semierect setae. Metepisternum partially visible in lateral view; conspicuously clothed with recumbent, pale setae. Metaventrite globose; strongly convex; surface shiny; longitudinal depression present; metaventral process absent.

Elytra: Convex, robust; humeri indicated, gradually expanding toward elytral apex, then abruptly narrowing behind elytral fourth; conspicuously vested with fine, pale, recumbent and semi-recumbent setae, vestiture density is reduced on anterior fourth where elytral disc acquires a testaceous tone; elytral disc rugulose throughout the surface; elytral apices rounded, moderately dehiscent; epipleural fold complete, narrowing toward apex.

Legs: Femora swollen anteriorly; surface shiny, smooth, very finely rugulose. Tibiae longitudinally rugulose; two tarsal denticles, outer denticle digitiform, interior denticle triangular in shape.

Abdomen: Ventrites 1-4 broadly convex, smooth, subquadrate, feebly punctate, not depressed laterally. Fifth visible ventrite convex, shiny and moderately depressed medially; lateral margins subparallel; posterior margin broadly truncate. Sixth ventrite triangular in shape; small; moderately excavated; shiny; feebly punctate; conspicuously broader than long; lateral margins strongly oblique, feebly arcuate; posterior margin small, broadly, deeply emarginate; posterolateral angles broadly rounded (Fig. 5G). Fifth tergite subquadrate; strongly convex; rugulose; feebly punctate; posterior margin truncate. Sixth tergite subquadrate; rugulose; wider than long; surface moderately convex; coarsely punctate; lateral margins oblique, posterior margin truncate; posterolateral angles rounded. Sixth tergite extending beyond apical margin of sixth ventrite, fully covering sixth ventrite in dorsal view.

Aedeagus: Not available.

Female variation: Females of *Callotillus bahamensis* can be distinguished from male specimens based on the antennal structure and the shape of the last abdominal segment. The females have antennomeres 4-9 moderately serrate (Fig. 3I), rather than strongly serrate, as observed in males (Fig. 3G). Additionally, the last ventrite and the last tergite are subquadrate in shape (Fig. 5H), and not emarginate, as in males (Fig. 5G).

##### Material examined.

ALLOTYPE: 1 female: South Bimini Island, Bahamas, B. W. I., VI-1951, M. Cazier and C and P Vaurie, handwritten red label, allotype depository: SMNH.

##### Additional material examined (N=2).

1 male, 1 female: Cayman, Little Cayman, 3 km SE of Spot Bay, 27-V-2009, R. Turnbow.

##### Remarks.

In her original description, Vaurie (1952) indicated that *Callotillus bahamensis* is most closely related to *Callotillus crusoe*. After examination of specimens of *Callotillus bahamensis*, this species is most similar to *Callotillus eburneocinctus*. Based on Wolcott’s description (1921), we place *Callotillus crusoe* within *Neocallotillus*. Examination of material of *Callotillus crusoe* will be essential to clarify the status of this rare species.

#### Callotillus eburneocinctus

Taxon classificationAnimaliaColeopteraCleridae

Wolcott, 1911

##### Holotype depository.


 United States National Museum of Natural History
(USNM). Holotype locality: Key West, Monroe Co., Florida.

##### Distribution.

USA: FL (Fig. 8E).

##### Differential diagnosis.

*Callotillus eburneocinctus* is most similar to *Callotillus bahamensis*. Characters to distinguish these species appear in the diagnosis section of *Callotillus bahamensis*.

##### Redescription.

Form: Body moderately elongate, robust; head and pronotum somewhat slender; elytra gradually expanded toward apex, then abruptly narrowing behind distal fourth. Color: head, antennae, mouthparts, pronotum, and anterior half of elytral disc testaceous to rufous; legs brunneous; distal end of mandibles, abdomen and posterior half of elytral disc fuscous; thorax bicolored, metaventrite anteriorly and internally ferrugineous, posteriorly and distally fuscous; each elytron with a transverse, median, pale fascia that runs from the epipleural fold to the elytral suture, this band may be protruded in most individuals (Fig. 1H).

Head: Including eyes not wider than pronotum; eyes small, taller than wide, not bulging laterally, finely faceted, strongly, sub-triangularly emarginate; surface integument corrugate; antennal notch located in front of antennal emargination; frons not bi-impressed. Antennae of males consisting of 10 antennomeres; second antennomere short, robust; third antennomere about 2× the length of previous antennomere, moderately serrate; fourth antennomeres slightly longer than third antennomere; antennomeres 4-9 about the same length, strongly serrate; last antennomere elongate, about 2× the length of ninth antennomere, slightly ovoid in shape, laterally compressed (Fig. 3H). Anterior portion of clypeus narrow, approximately 2× the length of eye emargination (Fig. 6B).

Thorax: Pronotum globose, as broad as long; surface rugulose, profusely, finely punctate; punctations narrow, shallow; sides subparallel in dorsal view, then abruptly constricted on posterior fourth; disc strongly convex; anterior transverse depression and subbasal tumescence absent. Prosternum wider than long; surface smooth, rugulose, glabrous. Mesoventrite smooth, shiny, glabrous; surface very finely punctate. Metaventrite strongly convex; surface smooth, feebly, finely punctate; longitudinal depression present; metaventral process absent.

Elytra: Robust; humeri indicated, gradually expanding toward elytral apex; surface convex on first third, then moderately depressed on second third, and conspicuously convex again on last third; a pair of long, stiff, erect tuft of dark setae located on the anterior fourth each elytron; surface of elytral disc rugulose; sculpturing absent; elytral apices rounded, moderately dehiscent; epipleural fold complete, narrowing toward apex.

Legs: Femora moderately swollen; surface shiny, smooth. Tibiae longitudinally rugulose; two tarsal denticles, outer denticle digitiform in shape, interior denticle triangular.

Abdomen: Ventrites 1-3 broadly convex, smooth, shiny, subquadrate, feebly punctate, not depressed laterally. Fourth ventrite moderately punctate, medially depressed. Fifth ventrite shiny, strongly excavated; lateral margins subparallel; posterior margin broadly, shallowly emarginate. Sixth ventrite triangular in shape; small; moderately excavated; feebly punctate; conspicuously broader than long; lateral margins strongly oblique, moderately arcuate; posterior margin shallowly, narrowly emarginate; posterolateral angles rounded (Fig. 5I). Fifth tergite subquadrate; moderately, coarsely punctate; posterior margin truncate. Sixth tergite subtriangular in shape; feebly convex; wider than long; surface moderately punctate; lateral margins strongly oblique, moderately arcuate; posterior margin very shallowly, narrowly emarginate; posterolateral angles broadly rounded. Sixth tergite extending beyond apical margin of sixth ventrite, fully covering sixth ventrite in dorsal view.

Aedeagus: Phallobasic apodeme present; phallus with copulatory piece feebly swollen at apex, petiolate; phallobase subparallel; phallic plate armed with one irregular row of denticles; intraspicular plate present, elongate; phallobasic lobes free; tegmen complete, fully covering phallus; phallobasic lobes rounded distally; phallobasic apodeme moderately short, expanded distally; endophallic struts short, robust distally (Fig. 7D).

Female variation: The structure of the antennae will help to differentiate females of *Callotillus eburneocinctus* from males. Females have the third antennomere feebly serrate and the antennomeres 4-9 are moderately serrate (Fig. 3J), males have the third antennomere moderately serrate and antennomeres 4-9 are strongly serrate (Fig. 3H). Females also have the sixth ventrite subquadrate in shape and the posterior margin is broadly truncate (Fig. 5J), males have this segment subtriangular in shape with the posterior margin narrow and feebly emarginate (Fig. 5I).

##### Material examined (N=35).

2 males, 3 females: Monroe Co., FL, Big Pine Key, 17-IV-1978, E. Giesbert; 1 male, 1 female: Monroe Co., FL, Everglades Nat. Park, Flamingo, 16-V-1991, R. Morris; 1 male, 2 females: FL, Miami, 13-VI-1963, B. K. Dozier; 1 female: FL, Tavernier, on Key Largo, 19-VI-1970, beating *Laguncularis racemosa* (L.), G. H. Nelson; 2 males, 3 females: FL, Miami, Virginia Key, 23-VI-1970, beating *Conocarpus erecta* L., G. H. Nelson. 1 male: Miami-Dade Co., FL, Miami, 25-VI-1965, B.K. Dozier; 4 specimens: FL, No Name Key, 29-V-1997, R. Turnbow, ex. *Metopium toxiferum* L., emerged 31-III-1979, R. Turnbow; 1 specimen: FL, Sugarloaf Key, 2-V-2000, 30-V-1997, R. Turnbow; 4 males, 9 females: Monroe Co., FL, Big Pine Key, reared from wood, E. Giesbert.

## Supplementary Material

XML Treatment for Neocallotillus

XML Treatment for Neocallotillus elegans

XML Treatment for Neocallotillus crusoe

XML Treatment for Neocallotillus intricatus

XML Treatment for Callotillus

XML Treatment for Callotillus bahamensis

XML Treatment for Callotillus eburneocinctus
